# Global Rietveld Refinement

**DOI:** 10.6028/jres.109.011

**Published:** 2004-02-01

**Authors:** Kenneth Shankland

**Affiliations:** ISIS Facility, Rutherford Appleton Laboratory, Oxfordshire OX11 0QX, United Kingdom

**Keywords:** global optimisation, powder diffraction, structure determination

## Abstract

Global optimisation methods of structure determination from powder diffraction data have risen to prominence in a relatively short space of time and they now constitute a key approach in the examination of polycrystalline molecular organic materials. A correctly formulated global optimisation approach may be regarded as a “global Rietveld refinement” that is capable of delivering accurate crystal structures from high-quality powder diffraction data. This paper focuses on how accuracy at all stages of a powder diffraction experiment impacts upon the overall structure solution process and particular attention is paid to assessing the degree of accuracy with which structures are returned from the global optimisation process.

## 1. Introduction

Faced with the challenge of applying traditional methods of crystal structure solution to powder diffraction data collected from molecular organic compounds, a limited number of options are currently available. One option is to seek to obtain a set of structure factors that is single-crystal-like in terms of quality and then apply conventional direct methods of structure solution. For anything other than structures containing strongly scattering atoms (e.g., S, Cl, Br) in small unit cells (up to say, a few hundred Å^3^ in volume), this task demands experimental approaches that exploit techniques such as differential thermal expansion or induced texture [1]. Alternatively, one can adapt direct methods of structure solution to take account of the inherent uncertainties in the structure factor magnitudes typically extracted from a powder diffraction pattern. Most productively, one can combine these approaches in order to maximise the chances of success.

The early 1990s saw numerous ingenious algorithmic developments in the areas of, for example, maximum entropy, direct methods and Patterson methods. However, by then, it was also clear that supplementing the available diffraction information with prior chemical knowledge of the compound under study was a powerful alternative (and sometimes complementary) approach [2–4]. In dealing with molecular organic compounds, one approach is particularly straightforward. Firstly, a three-dimensional representation of the compound of interest is created in Cartesian space using the known atom types and connectivity in conjunction with tabulated bond lengths, bond angles and bond torsion angles where appropriate. Those torsion angles that cannot be assigned accurate values in advance are simply assigned random values. Next, a trial crystal structure is constructed by randomly positioning and orienting this molecular model (by now, transformed to fractional coordinates) in the known unit cell, taking into account known space group information. After calculating diffraction data and comparing it against the measured diffraction data, the variable parameters of the model (typically, the molecular position, orientation and conformation) are adjusted in order to maximise the level of agreement between the observed and calculated data (i.e., minimise *χ*^2^), at which point the structure is solved if the global minimum in *χ*^2^ space has been located. However, despite its promise, this direct-space approach (so called, because the adjustable parameters lie in real rather than reciprocal space) was restricted in application to rigid or near-rigid molecules until only a few years ago. There were two main reasons for this. Firstly, many thousands of trial structure evaluations are typically required even when dealing with molecules with no internal degrees of freedom. As the calculation of even a single agreement factor based upon the full diffraction profile is, relatively speaking, a computationally intensive process, then the structure solution process is quite time consuming. Secondly, the search methods employed at the time (e.g. grid search) were not especially sophisticated.

Clearly, the ability to use intensities extracted from a diffraction profile offers a great opportunity for speeding up the structure evaluation process, i.e., the time-consuming peak shape and profile calculations need be performed only once, during the intensity extraction process. At the time though, it was widely believed that individual extracted intensities were too unreliable for this purpose due to the problem of reflection overlap. If, however, one uses the sums of intensities for groups of overlapping reflections [4] or the correlated integrated intensities extracted during a Pawley refinement of the diffraction data [5], this objection may be overturned. Accordingly, evaluation of each trial structure may be carried out using, for example:

$$\chi^{2}=\sum_{h}\sum_{k}\left[\left(I_{h}-c{\left|F_{h}\right|}^{2}\right){\left(V^{-1}\right)}_{hk}\left(I_{k}-c{\left|F_{k}\right|}^{2}\right)\right]$$ (1)

where *I_h,k_* is the extracted intensity from a Pawley refinement of the diffraction pattern, *V_hk_* is the covariance matrix from the Pawley refinement, *c* is the scale factor and *F_h,k_* is the calculated structure factor from the current trial structure. This approach is mathematically equivalent to the Rietveld method and for programs that implement it, such as DASH^1^ [7] the resultant increase in the rate at which structures can be evaluated is impressive. Taking the structure solution of hydrochlorothiazide from synchrotron powder diffraction data as an example (23 atoms, 204 reflections up to 1.5 Å resolution, 9726 points in the profile), DASH evaluates *ca*. 3500 trial structures per second running on a single processor 800 MHz Intel Pentium III PC.

The now commonly employed optimisation methods of simulated annealing and evolutionary algorithms (which include genetic algorithms) possess distinct advantages over the Monte-Carlo [2] and grid search [3–4] methods previously employed, in that they search the relevant parameter space in a much more efficient manner. It is the combination of these search algorithms with the aforementioned speed gains that have taken direct-space methods from a niche interest to a mainstream approach that is now at least competitive with direct methods of structure solution for powders in the field of molecular organic materials [6,8,9]. That global optimisation can now be considered routine for many powder diffraction problems is emphasised by the significant increase in the number of published structures solved in this way. For a more detailed explanation of the underlying principles of the global optimisation approach to structure solution and the many variants on the basic theme, see Shankland and David in [1].

## 2. Accuracy With Respect to Structure Determination

The route taken in deducing a crystal structure from powder diffraction data is only as strong as its weakest link. If, for example, the unit cell dimensions of the structure cannot be determined, then the structure solution process stalls at this stage. As increasingly complex structure determinations are tackled by powder diffraction, it therefore becomes imperative to pay particular attention to accuracy at all stages of the process.

### 2.1 Sample Preparation

The usual considerations of sample purity apply to any structure determination from powder diffraction data (SDPD) attempt, i.e., is the sample chemically pure and, if so, is it a single structure or a mixture of polymorphic forms? Whilst the presence of chemical impurities or multiple polymorphs does not preclude structure solution (see, for example, the determination of telmisartan form B in the presence a second solvated form, [10], or the determination of two forms of (CH_3_)_2_SBr_2_ in a mixed phase [1]) it is nevertheless a significant complicating factor. Indexing two unknown cells from a single diffraction pattern is a non-trivial task, and the process of Pawley or Le Bail fitting the pattern for two phases introduces correlations between overlapping reflections in the two sets of structure factors, in addition to the correlations present within a single set. Whilst recognising that, in many cases, it is not possible (or straightforward) to obtain a “pure” sample to work with, it is certainly the case that having a single-phase sample removes one complication from the structure solution process.

An additional complicating factor in the global optimisation approach to structure solution is that, in general, the full molecular connectivity of the molecule under study must be known if the SDPD is to be successful. This means that NMR, mass spectrometry, IR and elemental analysis information should ideally point to an unambiguous two-dimensional molecular formula that can then be translated into a three-dimensional model within the global optimisation program. If the input structure is incorrect, then the full crystal structure cannot be determined correctly. However, if the input structure is close to that of the correct structure, it may still be possible to interpret the resultant “incorrect” crystal structure in such a way as to lead to the correct structure. For example, during the structure determination of [(C_5_H_4_B(CH_3_))_2_Fe]-4,4 '-bipyridine polymer from synchrotron x-ray powder data [11], initial attempts to solve the structure resulted in crystal structures in which the basic repeat unit was not long enough to cross the unit cell and complete the polymeric structure, indicating an error in the input model. Upon checking, it was found that a one “B(CH_3_)_2_” unit had accidentally been omitted from the two-dimensional sketch upon which the input model was based. Upon insertion of this group into the model, a crystal structure was obtained in which the monomer unit was able to span the unit cell and form a polymeric chain, giving a satisfactory Rietveld refinement.

Other, less significant inaccuracies in the input molecular structure can sometimes be tolerated. For example, hydrogen atoms are frequently omitted from input models in order to simplify their construction or to speed up the calculation of structure factors by decreasing the number of contributing atoms that need to be considered. Such an omission is unlikely to hinder a structure determination unless it constitutes a significant part (for example, 20 %) of the overall scattering power of the molecule. Often, decisions about the correctness of a particular structural feature can be deferred to the structure completion stage, or structural ambiguities handled in a multi-solution approach. For example, both the *cis* and *trans* isomers of a molecule can be constructed and optimised independently against the diffraction data in order to determine which one is correct. The application of traditional “structure completion” methods such as Fourier recycling are still extremely valuable at the end of a global optimisation structure solution, as they can indicate the presence of features that the chemist failed to note or anticipate e.g. solvent of crystallisation.

### 2.2 Data Collection

It is difficult to collect accurate x-ray powder diffraction data to atomic resolution for the majority of molecular organic compounds, due to a combination of the Lorentz-polarisation factor and form-factor fall-off, compounded by a lack of strongly scattering atoms in the compounds. It has been known for several years now that employing a variable counting time (VCT) scheme, in which the “weak” diffraction data at higher angles is collected for much longer than the “strong” data at lower angles, confers significant benefits at the stage of Rietveld refinement. By the same token, in structure determination, where accurate structure factors (or sums of structure factors for overlapping reflections) are required, a VCT strategy can greatly enhance the chances of success. A simple and effective VCT strategy is to calculate the data collection time, *t*, for each particular 2*θ* value in the pattern using:

$$t(\theta )\propto (\sin\;\theta \;\sin\;2\;\theta )/\lfloor f_{\text{av}}^{2}(\theta )\;\exp(-2\;B_{\text{av}}\;\sin^{2}\;\theta /\lambda^{2})\rfloor$$ (2)

where *f*_av_ is a representative atomic scattering factor (e.g., carbon), *B*_av_ is an estimated overall Debye-Waller factor and *λ* is the incident wavelength.

**Fig. 1:**
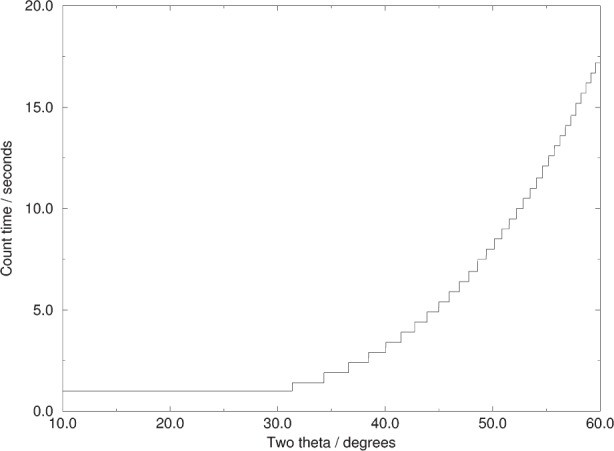
A variable count time scheme used during a data collection from a polycrystalline sample of the diuretic agent chlorothiazide. The original continuously variable scheme was approximated by 33 ranges of constant count time with a 0.5 s difference between each adjacent range. The minimum count time allowed was 1.0 s.

**Fig. 2:**
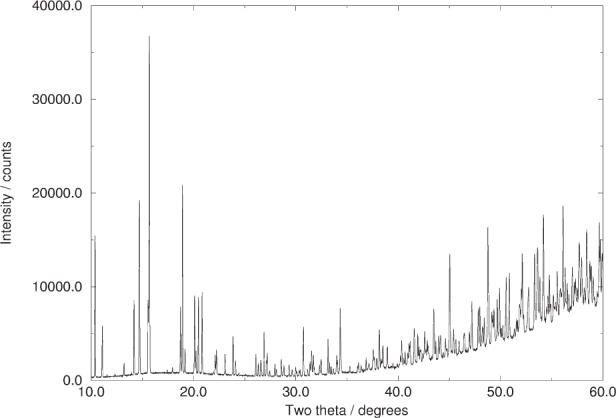
Raw powder diffraction data (i.e., as yet un-normalised with respect to the variable count times) collected from a polycrystalline sample of chlorothiazide on station 9.1 of the Daresbury SRS, using the variable count time scheme shown in Fig. 1. Note the excellent signal-to-background ratio at high angle where many of the strongest |*E*| values lie.

The resultant VCT scheme is shown in Fig. 1 for the case of a 6 h data collection from a sample of chlorothiazide at a wavelength of 1.1 Å, assuming a *B* value of 1 and setting the minimum count time to be 1 s. The benefits in terms of the corresponding diffraction data are clearly illustrated in Fig. 2. It is significant that 80 % of the data collection time was spent in the range 40° to 60° and that 75 % of the strong |*E*| values (|*E*| > 1.5) lie in this range. Use of the VCT scheme enabled a direct methods solution in which all the seventeen non-H atoms in the structure were identified from the top |*E*|-map [12].

The published literature contains many examples of structure solution problems where constant count times were used and it is not at all obvious why the authors did not take advantage of a VCT scheme. One suspects that the reasons for this are not particularly well-founded and that the popularity of the constant count time scheme is simply to do with the fact that it is “traditional”; in other words, “the way it has always been done”.

### 2.3 Profile Fitting

The ability to fit the shapes of individual diffraction peaks accurately has important implications not only for the final Rietveld refinement, but also for all stages of the structure determination process once the diffraction data has been collected. Many diffraction patterns can be adequately described using a Voigt (or pseudo-Voigt) peak shape and the increasing use of corrections for axial divergence [13] means that fitting asymmetry in peaks at low diffraction angles is no longer a problem.

#### 2.3.1 Indexing

Accurate fitting of low angle peaks returns the accurate peak positions that are essential in obtaining a good indexing solution. On a modern synchrotron powder diffraction beamline such as BM16 at the ESRF, the intrinsic accuracy of the diffractometer is such that high figures-of-merit for powder indexing solutions are the norm, even if the peak positions of the first twenty or so lines are simply estimated using a cursor. With data as good as this, accurate peak fitting can result in unexpectedly high figures-of-merit. For example, an F(40) value >2150 was obtained for the best cell corresponding to a diffraction data set collected on station BM16 from a proprietary pharmaceutical compound.

#### 2.3.2 Structure Factor Extraction

It is particularly important to have a good fit to the diffraction peaks during the stage of intensity extraction. Poorly fitted peaks lead to poor intensity estimates that then mislead structure solution attempts. Of particular importance here is the higher angle diffraction region, where it is difficult to distinguish the weaker Bragg diffraction features from the background “noise”. Correlations between the parameters of a refineable background profile and the refineable intensities in a Pawley or a LeBail fit can lead to inaccurate intensity estimates. The chances of this happening are significantly decreased if a VCT strategy has been employed and they can be decreased still further if the pattern being fitted is first carefully background subtracted, as the need to then simultaneously refine background parameters during the extraction step is eliminated.

#### 2.3.3 Space Group Determination

**Fig. 3:**
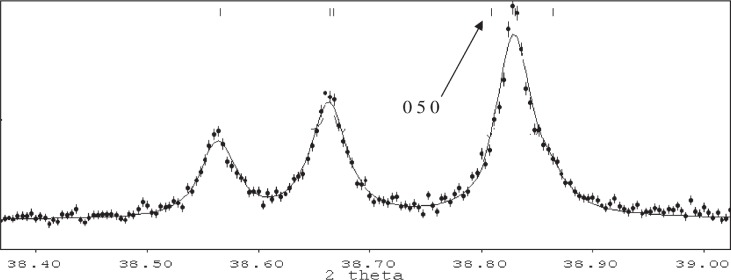
Part of an x-ray powder diffraction pattern collected from a sample of the diuretic agent hydrochlorothiazide (space group *P*2_1_) on beamline X7A of the NSLS at the Brookhaven National Laboratory. The 0 5 0 reflection lies slightly to the left of a relatively strong peak and so its presence / absence is difficult to establish by eye alone.

This is a stage of the structure determination process that is often considered to be straightforward but which stills relies heavily upon the intuition and ingenuity of the crystallographer, plus some knowledge of the relative frequencies of occurrence of the common space groups exhibited by organic materials. Often, the choice of space group will be clear, based on the size of the unit cell, the volume of the molecule and the presence or absence of certain diagnostic low-angle reflections. However, due to the problem of peak overlap, space group choices are frequently made on the basis of the presence or absence of only one or two diffraction peaks. Higher order systematic absences occurring at higher two-theta positions in the diffraction pattern are often obscured by other Bragg peaks and so cannot easily be factored into the decision making process (Fig. 3). A less subjective approach to the problem has been outlined by Markvardsen et al. [14]. In this approach, all the data (i.e., the extracted correlated integrated intensities from a Pawley fit to the diffraction data) are consulted and the probabilities of each of the possible extinction symbols (consistent with the crystal symmetry) relative to the extinction symbol possessing no systematic absences, is calculated. Typical output from such a calculation is shown in Table 1 for the monoclinic structure decaflouroquarterphenyl (DFQP). Extinction symbol *I*1*a*1 is consistent with space groups *Ia* and *I*2/*a*, and in the case of DFQP, the molecule is centrosymmetric and the correct space group is *I*2/*a*. Experience has shown that a good fit to the diffraction profile is a prerequisite to the successful application of this approach.

### 2.4 Structure Solution

Ultimately, the accuracy of crystal structures determined by global optimisation methods is determined by the quality of the diffraction data against which the crystal structure is finally refined. In that regard, global optimisation is no different from any other structure solution method. Of more interest to those involved in the structure determination process is the question “how accurate is the answer output from the global optimisation process?” That is to say, “how close is the optimised structure to the final refined structure?” Again, the quality of the diffraction data plays a significant role, both in terms of the resolution to which it has been collected and the quality of the Bragg peaks at the highest data resolution.

For the purposes of this discussion, assume that the molecular structure has been parameterised in terms of variable torsion angles only, i.e., as a series of rigid units connected by bonds around which those rigid units can rotate. In principle, a correctly formulated global optimisation approach to structure determination is equivalent to a “global Rietveld refinement”. The global optimisation algorithm locates, orients and folds the molecule of interest within the unit cell such that agreement between the observed and calculated diffraction data is maximised. If necessary, a semi-global optimisation algorithm (such as a simplex) or a local minimiser (such as conjugate gradient) can be employed in the same data / parameter space to improve the efficiency of the final step of locating the exact best minimum. Assuming that the global minimum has indeed been located, then the structure so obtained is the one that equates to a final rigid-body Rietveld refinement in which no other parameters are varied. Further improvements in the fit to the diffraction data can then be obtained only by the introduction of additional parameters to the model; for example, by employing a traditional Rietveld refinement in which the atomic positions are refined individually, or a restrained Rietveld refinement in which atomic positions are refined individually subject to a series of restraints that help to maintain chemical sense.

Here, only structures that have been obtained directly from a global optimisation structure solution process are considered. Comparisons are then made with solutions that have been obtained either from a single crystal or from a Rietveld refinement in order to gauge the level of accuracy that can be expected.

#### 2.4.1 Tetracycline Hydrochloride

**Fig. 4:**
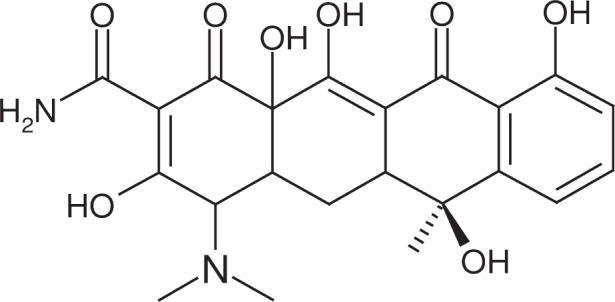
The molecular structure of tetracycline.

**Fig. 5:**
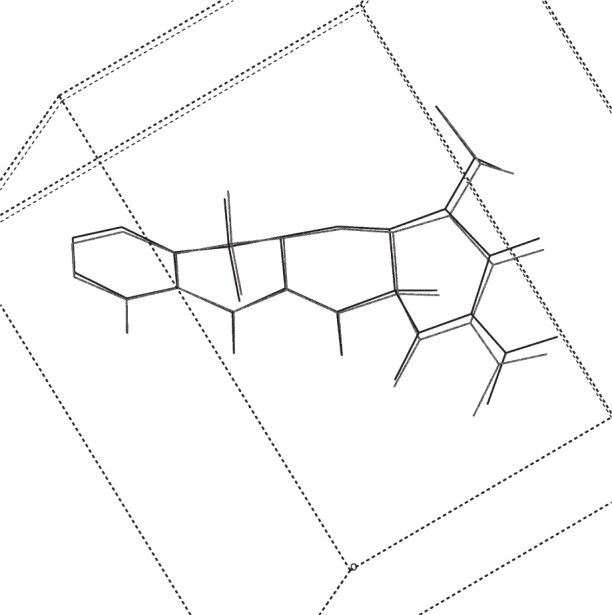
The crystal structure of tetracycline hydrochloride as determined directly from synchrotron powder diffraction data, overlaid upon the same structure as determined from a microcrystal at 150 K on station 9.8 at the Daresbury SRS. Hydrogen atoms and the chloride ion have been omitted for clarity.

**Fig. 6:**
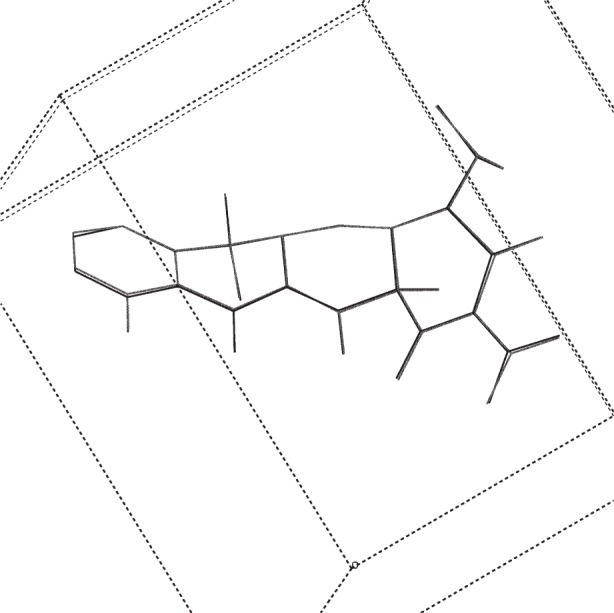
The Rietveld refined crystal structure of tetracycline hydrochloride, overlaid upon the microcrystal structure. Hydrogen atoms and the chloride ion have been omitted for clarity.

The determination of the crystal structure of the hydrochloride salt of the antibiotic compound tetracycline (Fig. 4) was set as a “blind test” of SDPD in 1998 [15], largely in response to the emergence of fast global optimisation methods of structure solution. Diffraction data collected from a polycrystalline sample of tetracycline hydrochloride (capillary, station 9.1 Daresbury SRS, *λ* = 0.692 Å, image plate detector, data range 2° to 40° 2*θ*) were posted on a web site along with the chemical formula (C_22_H_24_N_2_O_9_HCl), unit cell and space group (*a* = 10.981 Å, *b* = 12.853 Å, *c* = 15.733 Å, *P*2_1_2_1_2_1_) of the previously unsolved crystal structure. Participants were then invited to download the data and attempt to solve the crystal structure. Interestingly, the molecular connectivity of the molecule in question was not supplied. This is not a problem for direct or Patterson methods of solution but is a significant problem for global optimisation methods, as they rely upon knowing the molecular connectivity in advance. However, a simple search of a chemical reagent catalogue showed that the chemical formula of the molecule concerned matched that of tetracycline hydrochloride, suggesting that this was a likely candidate for the crystal structure to be determined. Using the supplied unit cell and space group, 594 correlated integrated intensities were extracted from the diffraction data in the range 3° to 30º 2*θ* by means of a Pawley fit, achieving an *R*_wp_ value = 2.3 %. Thereafter, an internal coordinate description of the positively charged tetracycline ion was constructed using the crystal structure of tetracycline as a prior source of the molecular topology. In this way, the molecule was parameterised as a rigid fragment with only two optimisable torsion angles connecting the –N(CH_3_)_2_ group and the amide group to the fused ring system. The position, orientation and conformation of the tetracycline ion and the position of the chloride counter-ion were then optimised against the extracted correlated integrated intensities using a simulated annealing technique implemented in a computer program running on a 433 MHz DEC Alpha Personal Workstation. Several structure solution runs were performed and solution times varied from 26 s to 600 s. Each run converged to give the same answer and was characterised by a rapid fall in the correlated integrated intensities *χ*^2^ value from starting values of around 7000 to finishing values of around 300. The solution thus obtained was found to be very close to the single crystal structure that was subsequently revealed by the test organisers (Fig. 5). The average separation between the positions of the non-H atoms is only 0.191 Å, with minimum and maximum deviations of 0.041 Å and 0.544 Å, respectively. Slack constrained Rietveld refinement against the full diffraction data range resulted in an improved fit to the data as a result of some changes in the previously fixed molecular topology of the fused ring system (Fig. 6). A final *R*_wp_ value of 2.9 % was obtained for the Rietveld fit.

#### 2.4.2 Capsaicin

**Fig. 7:**
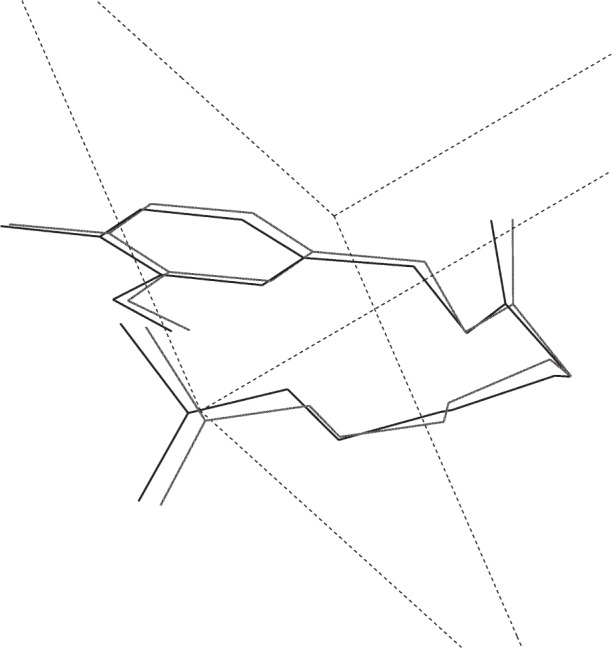
The crystal structure of capsaicin as determined directly from synchrotron powder diffraction data collected on BM16 at the ESRF, overlaid upon the same structure as determined from a single crystal on a laboratory diffractometer. Hydrogen atoms have been omitted for clarity.

The molecular crystal structure of capsaicin, the hot component of chilli peppers, was solved directly from synchrotron powder diffraction data alone, using the same simulated annealing procedure outlined in the previous section [6]. The internal coordinate description of the molecule was constructed using standard bond lengths, bond angles and bond torsions where appropriate. The level of agreement between the crystal structure obtained directly from the simulated annealing and the subsequently determined single crystal structure is excellent (Fig. 7) though a small degree of preferred orientation (discovered during subsequent restrained Rietveld refinement) precluded even better agreement.

#### 2.4.3 Promazine Hydrochloride

**Fig. 8:**
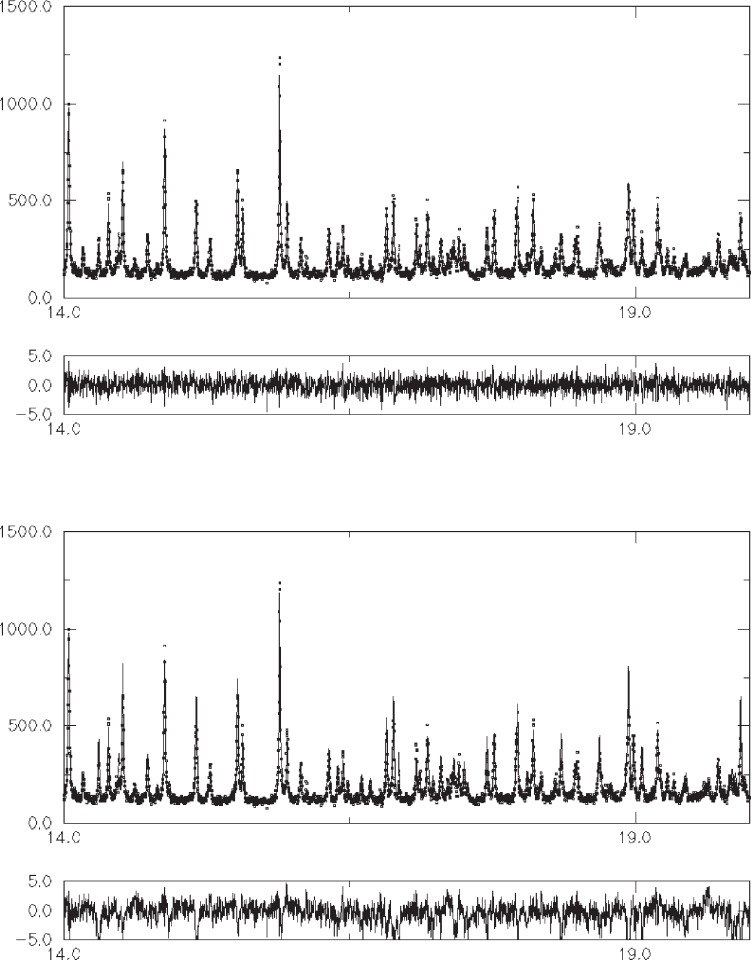
(a) A profile plot of the Pawley fit to synchrotron x-ray powder diffraction data collected from a sample of promazine hydrochloride on BM16 at the ESRF, *λ* = 0.6528 Å. (b) A profile plot of a scale-factor-only Rietveld refinement of the crystal structure of promazine hydrochloride as output directly from a simulated annealing procedure. In both plots, the calculated points are joined by a solid line whilst the observed points are represented by small open circles. Difference divided by e.s.d. plots are also shown.

The molecular crystal structure of the tranquilliser promazine hydrochloride was solved directly from synchrotron powder diffraction data alone, using the same procedure outlined for capsaicin. It is clear from Fig. 8 there is little difference between the model-independent Pawley fit to the data and the fit to the data given by a scale-factor-only refinement of the crystal structure output from the simulated annealing procedure. The level of agreement between observed and calculated data, particularly at higher angles, confirms that the crystal structure has been determined with a good degree of accuracy.

#### 2.4.4 Uridine

**Fig. 9:**
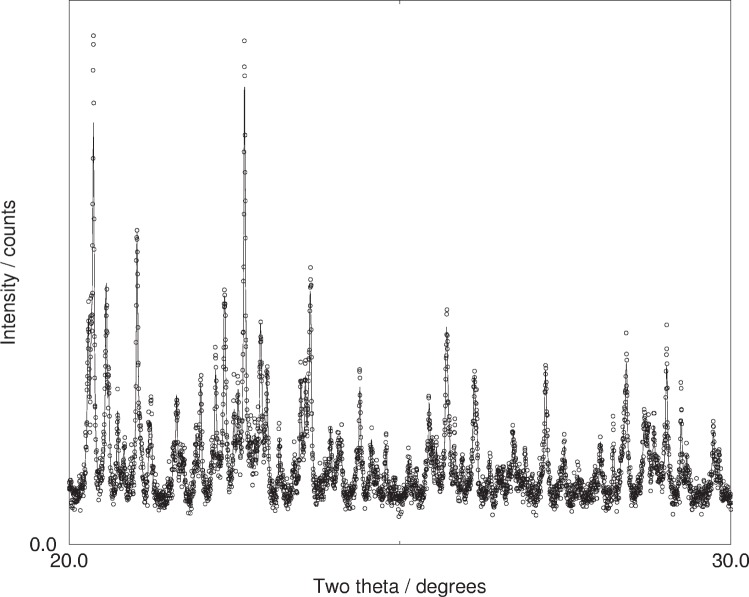
A profile plot of a scale-factor-only Rietveld refinement of the crystal structure of uridine as output directly from a simulated annealing procedure. The diffraction data were collected on BM16 at the ESRF. The calculated points are joined by a solid line whilst the observed points are represented by small open circles.

Another example of an excellent fit to the data at high angle obtained from the structure output direct from the simulated annealing procedure is shown in Fig. 9. The crystal structure in question is that of uridine, which contains two molecules in the asymmetric unit.

#### 2.4.6 Famotidine Form B

**Fig. 10:**
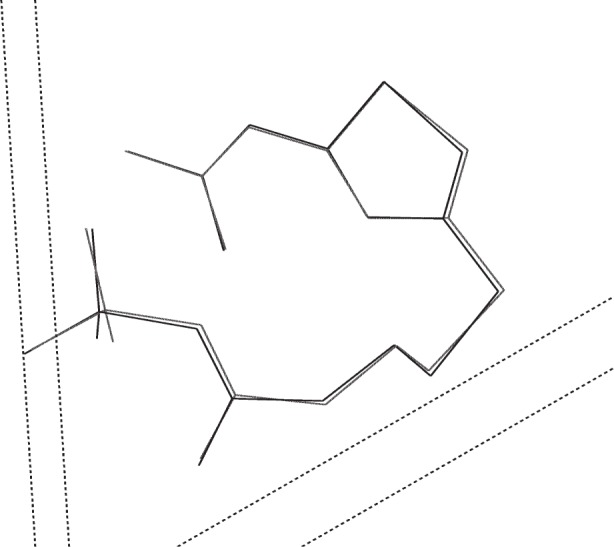
The crystal structure of famotidine (form B) as determined directly from synchrotron powder diffraction data collected on BM16 at the ESRF, overlaid upon the same structure as determined from a single crystal on a laboratory diffractometer. Hydrogen atoms have been omitted for clarity.

**Fig. 11:**
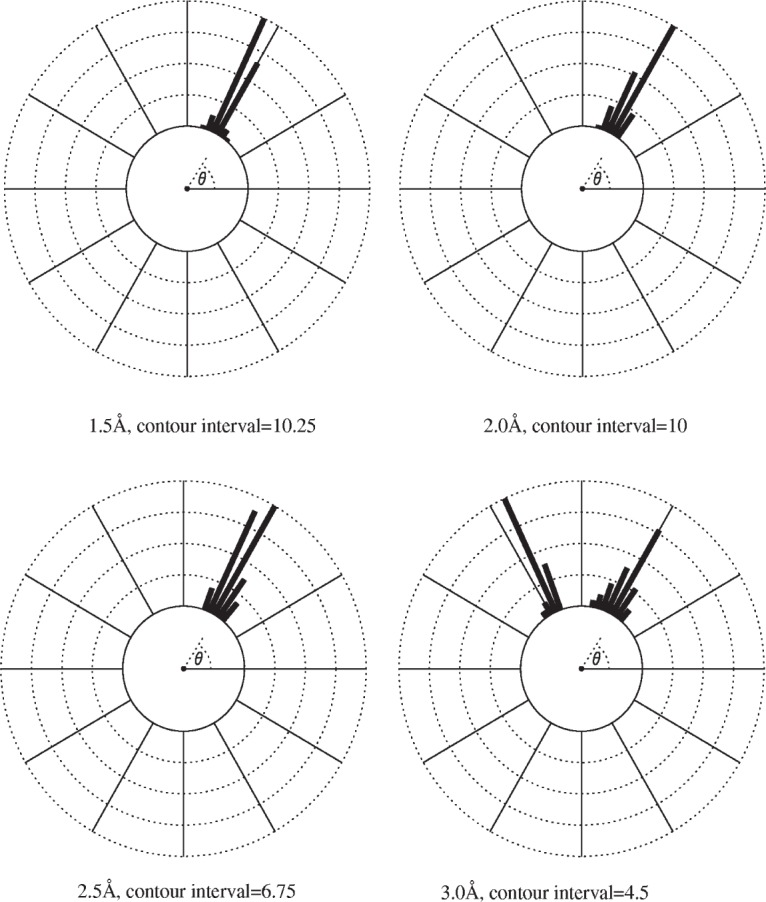
Polar plots of distributions of a single torsion angle within the famotidine molecule of the asymmetric unit. The value determined from the single crystal structure is 62.7°. The contours are in “number of solutions obtained”.

The crystal structure of famotidine form B was first solved from synchrotron x-ray powder diffraction data in 1998 using simulated annealing. Yet again, the correct solution (Fig. 10) is in excellent agreement with a subsequently determined single crystal structure. As a moderately complex organic crystal structure that can be solved relatively easily, it has since served as an excellent structure for evaluating the effects of varying algorithmic, chemical and crystallographic variables in the simulated annealing procedure. For a full discussion of the variables investigated, see Shankland et al. [16]. One of the most important findings of this work is the effect of diffraction data resolution upon the accuracy of the structure determination. Eighty simulated annealing solutions were obtained from each of four data sets truncated to the following resolutions: 1.5 Å, 2.0 Å, 2.5 Å, and 3.0 Å. The crystal structures from the successful runs (“success” meaning that each run reached a particular pre-set *χ*^2^ value) were then analysed and distributions of each of the optimisable torsion angles within the famotidine molecule calculated. Fig. 11 shows this distribution for one such torsion angle, whose value in the single crystal structure is 62.7°. At data resolutions as low as 2.5 Å, the distribution of torsion angles is always centred on the correct value, albeit with increasing spread as the resolution is decreased from 1.5 Å. At 3.0 Å, the distribution is bimodal. The results indicate that for a molecule of the complexity of famotidine, data should be collected to at least 2.5 Å resolution for reliable structure determination.

### 2.5 Accuracy of Input Structures

**Fig. 12:**
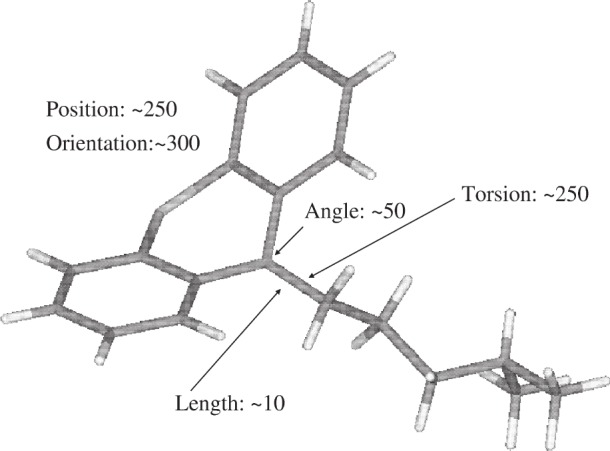
The molecular structure of promazine. The numbers on the diagram represent approximate “impact factors” for several variable parameters upon the correlated integrated intensities *χ*^2^ value obtained during a structure solution run of promazine hydrochloride. For example, varying the central bond torsion over the range 0° to 360° affects the fluctuations in *χ*^2^ by a factor of 25 more than varying the length of the central bond within chemically sensible (±0.1 Å) bounds. The impact of varying the position of the chloride ion (not shown) is ≈100.

**Fig. 13:**
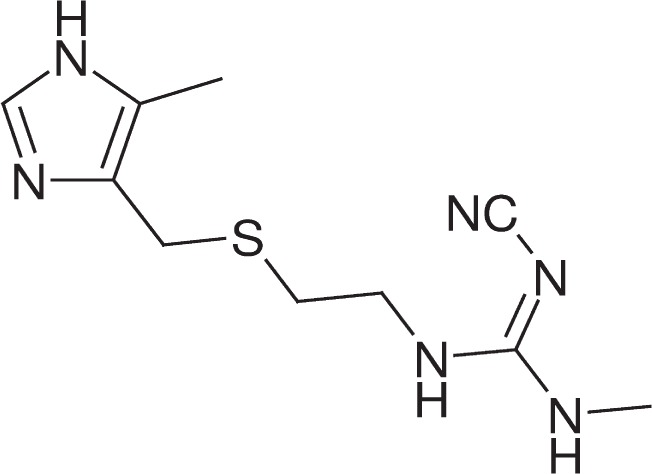
The molecular structure of cimetidine.

In sec. 2.4, only examples of structure determination in which the molecules under study had been parameterised in terms of a series of connected rigid bodies were shown. The justification for this approach is exemplified in Fig. 12, which shows the effective “fluctuations” in the correlated integrated intensities *χ*^2^ values seen as a result of varying certain key structural parameters during the structure solution of promazine hydrochloride. It is clear that it is the position, orientation and conformation of strongly scattering fragments that have the greatest impact upon the structure solution process, when parameters are varied within chemically sensible bounds. That is not to say that optimisation of bond lengths and bond angles may not be important in circumstances where their values are not known with sufficient accuracy in advance but, in the vast majority of circumstances, a “connected rigid body” parameterisation is likely to be effective. Furthermore, the accuracy achieved with such an approach will, in all likelihood, exceed that justified by the powder diffraction data alone. Nevertheless, a very accurate starting structure can help the structure solution process, if high quality diffraction data are available. This is illustrated in Table 2, which shows values of *χ*^2^ obtained from repeated DASH crystal structure solutions of cimetidine (Fig. 13) using a series of increasingly accurate input models. Successful structure determinations are indicated by the “*”. The energy-minimised model yields significantly better *χ*^2^ values than the model constructed simply using standard bond lengths and bond angles, although the structure solution success rate was unchanged in this small set of repeat runs.

### 2.6 Conclusions

It is certainly true to say that global optimisation methods of structure determination from powder diffraction data are now competitive with direct methods, at least for the case of molecular organic compounds. Their effectiveness reflects their ability to incorporate prior chemical knowledge in the form of the known connectivity of the molecule under investigation. They find particular utility in cases where direct methods of structure solution currently have difficulty, i.e., with low-resolution data, poor quality data, and “equal-atom” structures.

Ironically, it is now often easier to solve a moderately complex organic crystal structure to a chemically sensible answer than it is to refine it to “publication quality”. Powder diffraction is entering an era in which, the “acceptance criteria” for publication need to be carefully reconsidered, if useful crystal structures are to find their way into the public domain.

SDPD can be regarded as routine *in general*, although there are many *specific cases* within the current bounds of structural complexity where structure solution remains frustratingly difficult. If the range of applicability of SDPD is to be extended beyond the current bounds, attention now needs to be focused on the basic information content of the powder diffraction pattern and on how it can be enhanced by experimental and algorithmic developments. As an added complication, the ability to solve large crystal structures has taken us to a region where large unit cells are the norm and powder indexing often becomes the limiting step. That said, the fact that very large unit cells can be indexed from powder diffraction data when the diffraction features are close to instrumental resolution [17] provides a basis for optimism. Of one thing we can be sure—the many remaining problems associated with the accurate determination of organic structures from powders will serve as a spur to some exciting developments in the near future.

## Figures and Tables

**Table 1:** Log—likelihood values

Extinction symbol	Log-likelihood value
*I*1*a*1	153.2
*I*1–1	129.1
*P* 1 21/*n* 1	60.2
*P* 1 21/*c* 1	59.6
*P* 1 *n* 1	57.2
*P* 1 *c* 1	56.6
*P* 1 21/*a* 1	56.2
*P* 1 *a* 1	53.2
*P* 1 21 1	3.0
*P* 1–1	0.0
*A* 1 *n* 1	−3619.2
*A* 1–1	−3641.0
*C* 1 *c* 1	−4218.8
*C* 1–1	−4248.0

**Table 2:** *χ*^2^ values from DASH runs

Cimetidine input model	*χ*^2^ values from DASH runs
Not energy minimised	112*, 127*, 130*, 148, 226
Energy minimised	80*, 86*, 87*, 210, 214
Single crystal	64*, 73*, 82*, 87*, 90*

## Footnotes

1 Certain commercial equipment, instruments, or materials are identified in this paper to foster understanding. Such identification does not imply recommendation or endorsement by the National Institute of Standards and Technology, nor does it imply that the materials or equipment identified are necessarily the best available for the purpose.
